# Atteinte isolée de l'oreille au cours du pemphigus vulgaris

**DOI:** 10.11604/pamj.2015.20.444.6875

**Published:** 2015-04-30

**Authors:** Madiha Mahfoudhi, Khaled Khamassi

**Affiliations:** 1 Service de Médecine Interne A, Hôpital Charles Nicolle, Tunis, Tunisie; 2 Service ORL, Hôpital Charles Nicolle, Tunis, Tunisie

**Keywords:** Oreille, pemphigus vulgaris, immunofluorescence directe, ear, pemphigus vulgaris, direct immunofluorescence

## Image en medicine

Le pemphigus vulgaris est une maladie dermatologique chronique d'origine auto-immune. Il atteint la peau ou les muqueuses avec des lésions à type de bulles ou de croûtes. Il fait partie des maladies appelées dermatoses bulleuses auto-immunes. La fréquence de l'atteinte otorhinolaryngologique n'est pas précisée au cours du pemphigus vulgaris. Une atteinte isolée de l'oreille est rare. Patiente âgée de 67 ans, hypertendue, a consulté pour des lésions cutanées au niveau de l'oreille gauche. L'examen physique a objectivé des lésions érythémateuses et croûteuses étendues au niveau de la région prétragienne et du pavillon de l'oreille gauche ainsi que des érosions du conduit auditif externe. Le reste de l'examen était sans particularités. Le bilan biologique n'a pas trouvé de syndrome inflammatoire biologique. Le bilan infectieux était négatif. Le bilan immunologique (anticorps anti-nucléaires, ANCA, anticorps anti-phospholipides) était aussi négatif. L'examen anatomopathologique et en immunofluorescence directe a conclut à un pemphigus vulgaris. Le traitement s'est basé sur une corticothérapie par voie générale. L’évolution était marquée par une régression partielle des lésions.

**Figure 1:**
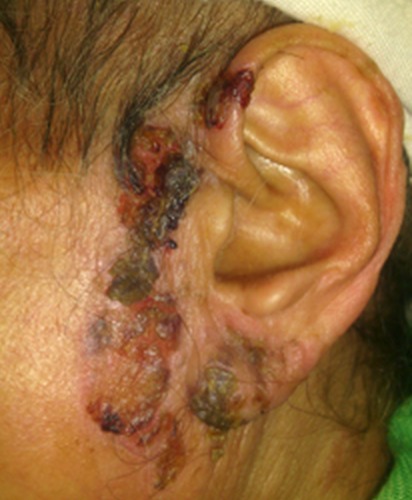
Lésions érythémateuses et croûteuses étendues au niveau de la région prétragienne et du pavillon de l'oreille gauche

